# Transcriptional Activation of REST by Sp1 in Huntington's Disease Models

**DOI:** 10.1371/journal.pone.0014311

**Published:** 2010-12-14

**Authors:** Myriam Ravache, Chantal Weber, Karine Mérienne, Yvon Trottier

**Affiliations:** Institute of Genetics and Molecular and Cellular Biology (IGBMC), Institut National de Santé et de Recherche Médicale (INSERM) U964/Centre National de Recherche Scientifique (CNRS) UMR 1704/Université de Strasbourg, Illkirch, France; Brigham and Women's Hospital, Harvard Medical School, United States of America

## Abstract

In Huntington's disease (HD), mutant huntingtin (mHtt) disrupts the normal transcriptional program of disease neurons by altering the function of several gene expression regulators such as Sp1. REST (Repressor Element-1 Silencing Transcription Factor), a key regulator of neuronal differentiation, is also aberrantly activated in HD by a mechanism that remains unclear. Here, we show that the level of REST mRNA is increased in HD mice and in NG108 cells differentiated into neuronal-like cells and expressing a toxic mHtt fragment. Using luciferase reporter gene assay, we delimited the REST promoter regions essential for mHtt-mediated REST upregulation and found that they contain Sp factor binding sites. We provide evidence that Sp1 and Sp3 bind REST promoter and interplay to fine-tune REST transcription. In undifferentiated NG108 cells, Sp1 and Sp3 have antagonistic effect, Sp1 acting as an activator and Sp3 as a repressor. Upon neuronal differentiation, we show that the amount and ratio of Sp1/Sp3 proteins decline, as does REST expression, and that the transcriptional role of Sp3 shifts toward a weak activator. Therefore, our results provide new molecular information to the transcriptional regulation of REST during neuronal differentiation. Importantly, specific knockdown of Sp1 abolishes REST upregulation in NG108 neuronal-like cells expressing mHtt. Our data together with earlier reports suggest that mHtt triggers a pathogenic cascade involving Sp1 activation, which leads to REST upregulation and repression of neuronal genes.

## Introduction

Huntington's disease (HD) is an autosomal dominant neurodegenerative disorder characterized by involuntary movements, psychiatric disturbances and cognitive decline. The pathology, which is characterized by a severe atrophy of the striatum, is due to an abnormal CAG repeat expansion in exon 1 of the *HD* gene that is translated into a polyglutamine (polyQ) expansion at the N-terminus of huntingtin (Htt) [1]. HD belongs to a group of 10 inherited neurodegenerative disorders, including 7 dominant spinocerebellar ataxias (SCAs), that are caused by polyQ expansion in specific disease proteins [2]. It is proposed that polyQ expansions confer toxic properties to mutant proteins by causing aberrant protein interactions and aggregation of polyQ-containing proteolytic fragments [3].

Increasing evidence indicates that mutant Htt (mHtt) disrupts the normal transcriptional program of disease neurons [4]. Early studies revealed that the levels of neurotransmitter receptors were altered in post-mortem brains of early HD [5], [6], [7], [8]. Expression profiling studies of HD mouse brains showed that hundreds of genes were deregulated, including a conspicuous decreased expression of neuronal specific genes in the striatum [9], [10], [11], [12]. Similarly, microarray analysis of HD post-mortem brains showed that the most extensive changes occurred in the striatum where neuronal specific genes expression was reduced [13]. Altered expression of neuronal specific genes was also observed in other polyQ disorders. In a SCA1 mouse model, specific genes involved in glutamate signaling functions were deregulated in Purkinje cells, one major target of the disease [14]. More strikingly, in a mouse model recapitulating the retinal degeneration affecting SCA7 patients, the retinopathy correlated with a global repression of genes specifically involved in photoreceptor function and morphogenesis, suggesting an alteration of the neuronal differentiation program [15], [16].

The mechanisms accounting for neuronal gene repression in HD are unclear. mHtt was shown to alter the activity of a large number of regulators of transcription [4], [17]. Among these, Sp1 is one of the most studied. If several lines of evidence suggest that mHtt binds to Sp1 and causes a loss of Sp1 function on selective genes promoters [18], [19], [20], other studies reported that Sp1 protein level is increased in HD and that genetic or pharmacological approaches which decrease the level or activity of Sp1 have beneficial effect on HD mice [21], [22]. Therefore, the role of Sp1 in neuronal gene repression is unclear and warrants further investigation.

The activation of REST (Repressor Element-1 Silencing Transcription Factor) in HD has gained much attention in recent years. REST, also named Neuron Restrictive Silencer Factor (NRSF) or Repressor Binding to the X2 box (XBR), is a critical regulator of neuronal terminal differentiation. REST is thought to silence neuronal genes in non-neuronal cells. During neuronal differentiation, REST is progressively downregulated at the protein level in neural precursors and at the mRNA level in post-mitotic neurons [23]. In the adult brain, REST transcript is expressed at very low levels [24]. Importantly, aberrant expression of REST in differentiating neurons causes repression of neuronal genes and leads to abnormal neuronal phenotypes [25], [26].

REST binds to repressor element 1 (RE1), which is present at several hundreds sites in human and mouse genomes, and many REST regulated genes are essential for neuronal development and differentiation [27], [28]. In cellular and mouse models of HD, REST shows an increased occupancy on RE1 of specific gene promoters that correlated with transcriptional repression of the corresponding neuronal gene products [29]. Consistently, ChIP-on-chip experiments revealed a significant increased of REST binding on RE1 in postmortem HD brains. In particular, REST binds to the promoter of brain-derived neurotrophic factor (BDNF) that is downregulated in HD [30]. A dominant-negative form of REST restored the BDNF level in HD cells [29].

Zuccato *et al* [31] showed that Htt interacts with and retains REST in the cytoplasm of neuronal cells. They proposed that mHtt causes a loss of Htt retention function in HD, leading to the nuclear translocation of REST where it represses neuronal genes [31]. Other studies suggest that REST regulation is altered by polyQ toxicity. Embryonic-stem cells expressing the *Hprt* gene containing a CAG expansion were shown to undergo aberrant neuronal differentiation correlating with persistent REST expression [32]. The global level of REST proteins is increased in the brain of the R6/1 mouse model of HD [33]. Although REST can regulate its own expression level through a double negative feedback loop involving REST-dependent expression of a specific microRNA [34], the mechanism underlying REST upregulation in HD remains unclear.

Here, we show that REST mRNA is increased in the R6/2 mouse model and NG108 neuronal-like model of HD, and that REST upregulation occurs at the transcriptional level. Using a luciferase reporter gene assay, we delimited the REST promoter regions essential for mHtt-mediated REST upregulation and found that they contain Sp factor binding sites. Consistently, we detected by chromatin immunoprecipitation (ChIP) that Sp1 and Sp3 proteins bind the REST promoter in NG108 cells and R6/2 mouse brains. We provide evidence that Sp1 and Sp3 interplay to fine-tune REST transcription during neuronal differentiation. Importantly, we demonstrate that Sp1 contributes to mHtt-mediated REST upregulation in neuronal-like cells. Thus, our data reveal an unexpected relationship between two transcriptional regulators, Sp1 and REST, which have both been shown to contribute to HD pathogenesis.

## Materials and Methods

### Animal care and use

All experimental procedures for care and use of mice were performed according to agreements with the Departmental Direction of Veterinarian Services (Prefecture du Bas-Rhin, France No. C67-218-5) and IGBMC Animal Welfare Insurance (NIH, PHS No. A5100-01). Mice were euthanized and brain tissues were removed for molecular analysis. The HD R6/2 mice from the Jackson Laboratory (Bar Harbor, ME, USA) were maintained in a mixed CBAxC57BL6 genetic background [35].

### Culturing and differentiation of cell line

The neuroblastoma/glioma NG108-15 rt30 hybrid cell line were grown in Dulbecco's modified Eagle's medium (DMEM) supplemented with 10% foetal calf serum (FCS), 1 g/L D-glucose, 40 µg/ml gentamicin and 0,5 mg/ml G418 [36]. The cells were maintained in a humidified 5% CO_2_ atmosphere at 37°C. For neuronal differentiation, medium was replaced by differentiation medium composed of DMEM, 1% FCS, 1 g/L D-glucose, 40 µg/ml gentamicin, 10 µM forskolin and 100 µM IBMX (isobuthylmethylxanthine). During time course experiments, the medium was changed every 2 days.

### Plasmid constructs and direct mutagenesis

DNA from human BAC clone RP11-738E22 was used as template for amplification of the human REST promoter. The 5′UTR (−3390/+1) and the deletion regions of human REST promoter were amplified by PCR using specific sets of primers (supplemental Table S1) with the exception of regions A1 and A2 that were obtained by restriction enzyme digestion of deletion construct A. PCR fragments were subcloned into the pJET1 vector (GeneJET™ PCR cloning kit, Fermentas) then inserted upstream of the *luciferase* gene in the pGL3-basic vector using restriction enzyme digestion.

To create REST promoter regions in which mutations were introduced in the putative NF-kappa B binding sites, deletion constructs A, B and C were amplified by PCR using *Pfu* DNA polymerase (Agilent Technologies) and sets of primers, which contained mutated NF-kappa B binding sites (supplemental Table S2).

Nucleotide sequences of all the clones generated by PCR were confirmed by sequencing. The Sp1 expression vector, which is a pN3 vector containing the coding sequence for human Sp1, and the empty pN3 vector were generous gifts from Guntram Suske (Marburg, Germany). The pEBGN and pEBGN-Sp1 plasmids were kindly provided by Dr Gerald Thiel [37]. The expression vectors of Nter-15Q or Nter-142Q correspond to the pcDNA3.1 vector containing the coding sequence for the first 171 amino acids of human Htt with 15Q or 142Q, respectively.

### siRNA interference

siRNA was transfected into NG108 cells with Lipofectamine 2000 (Invitrogen) in 6-well plates following the manufacturer's instructions. For the prolonged knockdown in NG108 cells differentiated into neuronal-like cells for 6 days, siRNA were added every 2 days. The control siRNA (OR-003-NEG20) was purchased from Eurogentec. Mouse Sp1 and Sp3 siRNAs were purchased from Santa Cruz Biotechnology (sc-29488 for Sp1 and sc-36544 for Sp3) or Abgene LTD (M-040633-02 for Sp1 and M-040397-01 for Sp3).

### Transient transfection and luciferase reporter assay

Transfections were performed with Lipofectamine 2000 (Invitrogen) or FuGENE 6 (Roche) in 6-well plates following the manufacturer's instructions. For the experiments done with NG108 cells differentiated into neuronal-like cells, cells were transfected 24 h prior to differentiation. For the luciferase experiments, cells were cotransfected with reporter genes, beta-galactosidase expression vector as an internal control for normalization and different expression vectors. The final DNA concentration in all experiments was maintained constant by addition of empty expression vector. After 48 h incubation or after 6 days of neuronal differentiation, cells were harvested and assayed for luciferase and beta-galactosidase activity.

### Western blot analysis

Cells were lysed in buffer containing 50 mM Tris-HCl pH 7.4, 150 mM NaCl, 1 mM EDTA, 1% Triton X-100, protease and phosphatase inhibitors. They were incubated for 15 min on ice and centrifuged at 10 000 rpm for 10 min. Supernatants were collected and analyzed by SDS-PAGE. Primary antibodies anti-REST (ab21635, Abcam), anti-Synaptophysin (MAB5258, Chemicon), anti-Sp1 (sc-14027, Santa Cruz Biotechnology), anti-Sp3 (sc-644, Santa Cruz Biotechnology) and anti-beta-tubulin were used and revealed with appropriate peroxidase-conjugated secondary antibodies and ECL chemiluminescent reaction (Millipore).

### RNA extraction and quantitative real-time reverse transcription-PCR

Total RNA was purified with GenElute™ Mammalian Total RNA Miniprep Kit (Sigma-Aldrich) following the manufacturer's instructions. Reverse transcription (RT) was performed on 2 µg of total RNA using SuperScriptII (Invitrogen) according to the manufacturer's instructions. The RT products were used as template for quantitative real-time PCR using a Light-Cycler instrument (Roche). The sequences of PCR primers used for gene expression analysis are indicated in supplemental Table S3. All results were normalized using quantification of housekeeping genes (*36B4*, *Hprt* or *Gapdh*) with specific PCR primers.

### ChIP assay and quantitative real-time -PCR

Confluent undifferentiated NG108 cells in 10-cm dishes were treated with formaldehyde at a final concentration of 1% to cross-link nuclear proteins with genomic DNA. After incubation for 2 min at 37°C, the reaction was stopped by addition of glycine (125 mM) for 5 min at room temperature. Cells were washed twice with cold PBS containing protease and phosphatase inhibitors, harvested and centrifuged at 2000 rpm at 4°C for 4 min. The cellular pellet was lysed in buffer containing 50 mM Tris-HCl pH 8.1, 10 mM EDTA, 1% SDS and protease inhibitors. After incubation for 30 min on ice, genomic DNA was fragmented into 200–500 bp fragments by sonication using the Bioruptor instrument (Diagenode). After sonication, the sample was diluted in the IP buffer (16,7 mM Tris-HCl pH 8.1, 167 mM NaCl, 1,1% Triton X-100, 1,2 mM EDTA, 0,01% SDS, protease inhibitors). A fraction of the sample was conserved as input. The sample was shaken gently at 4°C for 2 h with protein A (Protein A Agarose/Salmon Sperm DNA, 16-157C, Upstate). After centrifugation at 1400 rpm at 4°C for 1 min, the supernatant was immunoprecipitated overnight at 4°C with the antibodies, anti-Sp1 (sc-14027, Santa Cruz Biotechnology) or anti-Sp3 (sc-644, Santa Cruz Biotechnology). Anti-GST antibody was used as a negative control. After incubating with protein A (Protein A Agarose/Salmon Sperm DNA, 16-157C, Upstate) at 4°C for 4 h, beads were sequentially washed with low-salt buffer (0,1% SDS, 1% TritonX-100, 2 mM EDTA, 20 mM Tris-HCl pH 8.1, 150 mM NaCl), high-salt buffer (0,1% SDS, 1% TritonX-100, 2 mM EDTA, 20 mM Tris-HCl pH 8.1, 500 mM NaCl), LiCl buffer (0,25 M LiCl, 1% Nonidet P-40, 1% sodium deoxycholate, 1 mM EDTA, 10 mM Tris-HCl pH 8.1) and twice with TE buffer (10 mM Tris-HCl, 1 mM EDTA, pH 8.0). The immunoprecipitated DNA-protein complex was eluted with 100 mM NaHCO_3_, 1% SDS. The cross-link was reversed by incubating at 65°C overnight with 200 mM NaCl. After incubating with proteinase K for 1 h at 45°C, DNA was purified and analyzed by quantitative real-time PCR using a Light-Cycler instrument (Roche). The primers used for ChIP analysis of mouse REST promoter regions are indicated in supplemental Table S4.

ChIP experiments using brain samples from R6/2 mice and wildtype littermates were performed as described in [38]. Briefly, each immunoprecipitation was performed in triplicates from whole brains of 3 different mice at 12 week of age. The tissue was cut into small fragments, fixed by adding 37% formaldehyde to a final concentration of 1% and incubated for 10 min at room temperature. Cross-linking was stopped by addition of glycine to 0.125 M. Tissue fragments were washed three times with cold phosphate-buffered saline and treated with sonication buffer (50 mM HEPES pH 7.9, 140 mM NaCl, 1 mM EDTA, 1% Triton X-100, 0.1% sodium dodecyl sulfate, 0.1% Na-deoxycholate) containing protease and phosphatase inhibitors. Tissue was then homogenized, and lysates were sonicated to obtain DNA fragments of 200 to 500 bp. Samples were centrifuged to pellet debris and an aliquot was taken for gel analysis and input. The soluble chromatin fraction was pretreated for 1 h at 4°C with protein A agarose/Salmon Sperm DNA −50% slurry- (Millipore). Samples were then incubated overnight at 4°C with anti-Sp1 (sc-14027, Santa Cruz Biotechnology) or anti-Sp3 (sc-644, Santa Cruz Biotechnology) antibodies. Protein A agarose/Salmon Sperm DNA was then added, and the mixture was incubated for 2 h at 4°C. Agarose beads were washed twice for 4 min with sonication buffer, twice for 4 min with wash buffer A (sonication buffer with 500 mM NaCl), twice for 4 min with wash buffer B (20 mM Tris-HCl, pH 8.0, 1 mM EDTA, à.25 M LiCl, à.5% NP-40, 0.5% Na-deoxycholate), and finally with Tris-EDTA (TE, pH 8.0). Immune complexes were eluted from the beads with 1% SDS in TE (pH 8.0) and protein-DNA cross-links were reversed by adding 200 mM NaCl and heating overnight at 65°C. After treatment with proteinase K for 2 h at 42°C, the samples were purified by phenol-chloroform-isoamyl alcohol extraction and precipitated with ethanol. One-fifteen of the immunoprecipitated DNA and 1% of the input DNA were quantified by real-time quantitative PCR.

### Search for transcription factor binding sites

Alignment of the human, mouse and rat *REST* gene promoters was performed using ClustalW and genomic sequences from GenBank accession numbers AB024498, AB024497 and NM_031788, respectively. Search for conserved transcription factor binding sites was performed by combining the PromAn, TFSearch and MatInspector programs [39], [40].

### Statistical analysis

All data are expressed as mean ± SEM, except if stated otherwise. We performed analysis of significance using a one-way ANOVA or two-way ANOVA tests followed by Student-Newman-Keuls Method or a Student's *t* test followed by Mann-Whitney test.

## Results

### REST expression is increased in Huntington's Disease models

**Figure 1:**
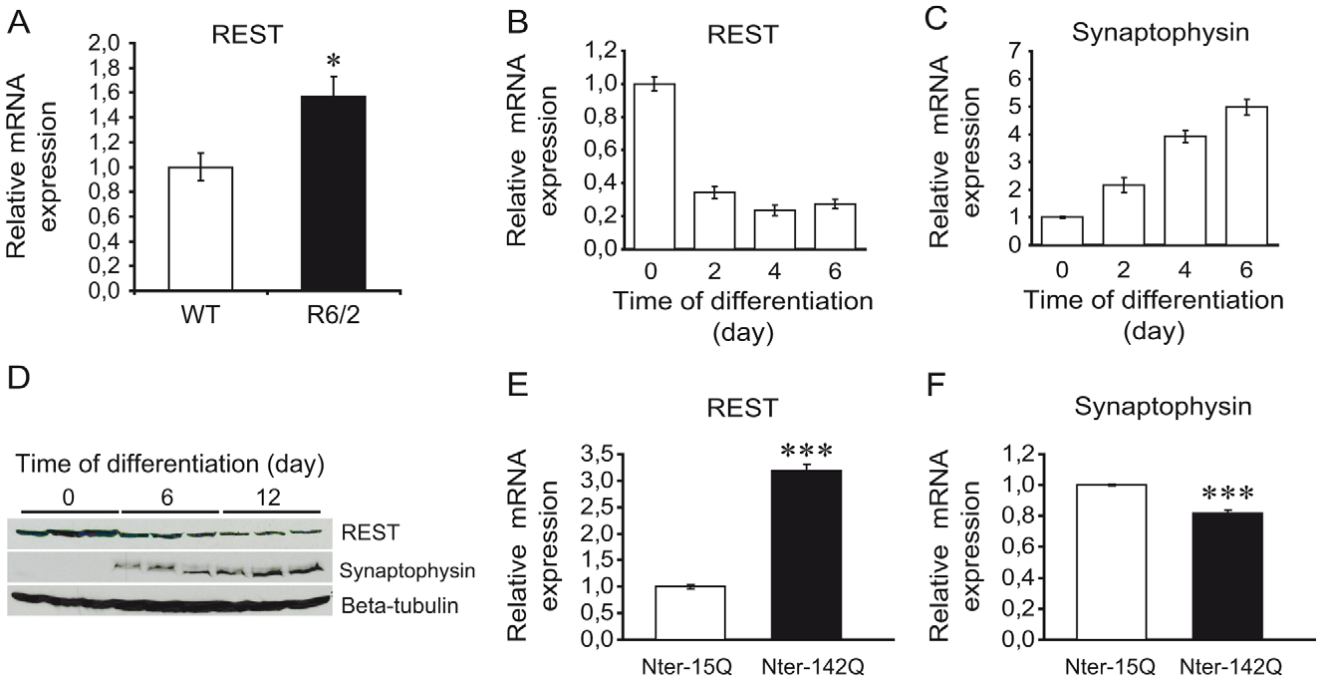
Increase of REST expression in HD mouse and cellular models. (A) mRNA level of REST in the striatum of 12-week-old wild-type (open bar) and R6/2 mice (solid bar). mRNA level was determined by quantitative RT-PCR. Each bar represents the mean value ± sem, *P<0,05 (WT: n = 4; R6/2: n = 4). (B,C) mRNA levels of REST (B) and Synaptophysin (C) in NG108 cells during 6 days of neuronal differentiation. mRNA levels were determined by quantitative RT-PCR. Each bar represents the mean value ± sem of at least 4 independent experiments performed in triplicates. (D) Protein levels of REST and Synaptophysin in NG108 cells during 12 days of neuronal differentiation. Protein levels were analyzed by Western blotting using beta-Tubulin as loading control. (E,F) mRNA levels of REST (E) and Synaptophysin (F) in 6-day differentiated NG108 cells expressing Nter-15Q (open bar) or Nter-142Q (solid bar). mRNA levels were determined by quantitative RT-PCR. Each bar represents the mean values ± sem of at least 4 independent experiments performed in triplicate. ***P<0,001.

Previous studies showed that expression of REST might be impaired in models of polyglutamine disorders [32], [33]. To thoroughly investigate this possibility and clarify the underlying mechanism, we analyzed mRNA levels of REST in two different HD models. First, we found that REST mRNA was significantly increased in the striatum of 12-week old R6/2 mouse model of HD compared to wild-type mice (Fig. 1A). Second, we studied the expression level of REST in a cellular model of HD. We used the neuroblastoma/glioma NG108 hybrid cell line transfected with mHtt and differentiated into neuronal-like cells by increasing the intracellular level of cAMP [36]. In untransfected NG108 cells, mRNA and protein levels of REST decreased with the acquisition of neuronal differentiation phenotype (Fig. 1B, D), consistent with the repressive role of REST in neuronal differentiation. Accordingly, decreased REST expression upon neuronal differentiation was correlated with increased mRNA and protein levels of Synaptophysin, a neuronal gene silenced by REST [41] (Fig. 1C, D). Compared to undifferentiated NG108 cells, REST mRNA was decreased by about 3-fold in 6-day differentiated neuronal-like cells, while Synaptophysin mRNA was increased up to 5-fold (Fig. 1B,C).

We then examined the effect of mHtt on REST expression in NG108 cells expressing an amino-terminal fragment (Nter) corresponding to the first 171 amino acids of human Htt with 142Q (thereafter called Nter-142Q) or with 15Q as control (Nter-15Q). mHtt fragment of this size has been shown to cause a neurological phenotype in mice [42]. After 6 days of neuronal differentiation, REST mRNA was dramatically increased in Nter-142Q compared to Nter-15Q expressing cells (Fig. 1E), while the latter did not differ from cells transfected with an empty expression vector (data not shown). The expression level of REST in Nter-142Q neuronal-like cells was comparable to that measured in undifferentiated NG108 cells (Fig. 1A), suggesting that Nter-142Q cells undergo aberrant neuronal differentiation. Consistent with this, the mRNA level of Synaptophysin was significantly decreased in Nter-142Q compared to Nter-15Q neuronal-like cells (Fig. 1F). Taken together, these data indicate that the Nter of mHtt induces aberrant expression of REST.

### Mutant huntingtin induces the transcriptional activation of REST promoter

**Figure 2:**
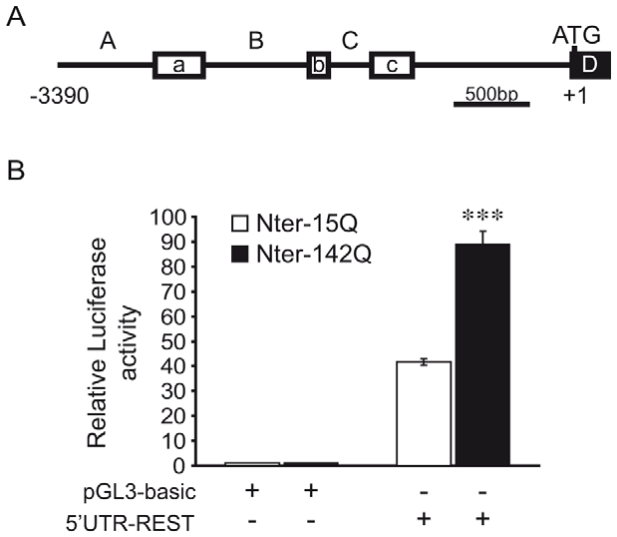
mHtt increases REST expression at the transcriptional level. (A) Schematic representation of the human *REST* gene promoter. Exons a, b and c (open boxes) are downstream of the alternative promoters A, B and C, respectively. These exons are alternatively spliced to exon D (solid box) which contains the translation initiation site, with the adenosine of the ATG start codon being set as +1. A -3390-bp/+1 fragment was cloned upstream of the *luciferase* reporter gene in the pGL3-basic vector, to generate the 5′UTR-REST vector. (B) Activity of *REST* gene promoter in 6-day differentiated NG108 cells expressing Nter-15Q or Nter-142Q. 5′UTR-REST or pGL3-basic vectors were cotransfected with either Nter-15Q (open bar) or Nter-142Q (solid bar) and beta-galactosidase expression vectors into NG108 cells. Cells were harvested after 6 days of neuronal differentiation to measure the luciferase and beta-galactosidase activities. Luciferase activity was normalized to beta-galactosidase activity and expressed as fold increase above the control pGL3-basic. Data are the mean values ± sem of 3 independent experiments performed in triplicate. ***P<0,001; Nter-15Q expressing cells *vs*. Nter-142Q expressing cells.

To clarify the mechanism whereby Nter of mHtt increases the expression of REST, we characterized the activity of the human REST promoter using a *luciferase* reporter gene assay. To do so, we cloned the −3390/+1 bp fragment of the human REST promoter upstream of the *luciferase* gene into the pGL3-basic vector. This generated the 5′UTR-REST vector. The REST promoter fragment contains three alternative promoters A, B and C, which drive, respectively, the expression of three upstream exons, a, b and c (Fig. 2A) [43], [44], [45]. Each upstream exon can be alternatively spliced to exon D, which contains the ATG translation initiation site of REST. In 5′UTR-REST vector, exon D was replaced by the *luciferase* gene cassette.

Nter-142Q and Nter-15Q cells were transfected with 5′UTR-REST vector and differentiated into neuronal-like cells, prior to the measurement of luciferase activity. Fig. 2B shows that luciferase activity was increased up to 2-fold in Nter-142Q compared to Nter-15Q neuronal-like cells. Thus, the Nter of mHtt induced the transcriptional activation of the human REST promoter.

### Identification of conserved transcription factor binding sites in the REST promoter

We reasoned that the transcriptional activation of REST in HD models might occur indirectly, since mHtt could alter the activity of specific transcription factor(s) involved in the regulation of *REST* gene expression. To identify such transcription factor(s), we performed a sequence alignment of human, mouse and rat REST promoters to unravel conserved elements. Using bioinformatics programs (see Materials and Methods), we identified several putative binding sites conserved between species. Among these, we found multiple binding sites for AP-1, NF-Kappa B and Sp factors (Supplemental Fig. S1), whose activities were shown to be altered in HD [18], [19], [46], [47]. These sites are abundant and dispersed along the promoter sequence, prompting us to narrow down the relevant promoter region(s) that mediate the effect of the Nter of mHtt.

### Identification of minimal REST promoter regions regulated by mutant huntingtin

**Figure 3:**
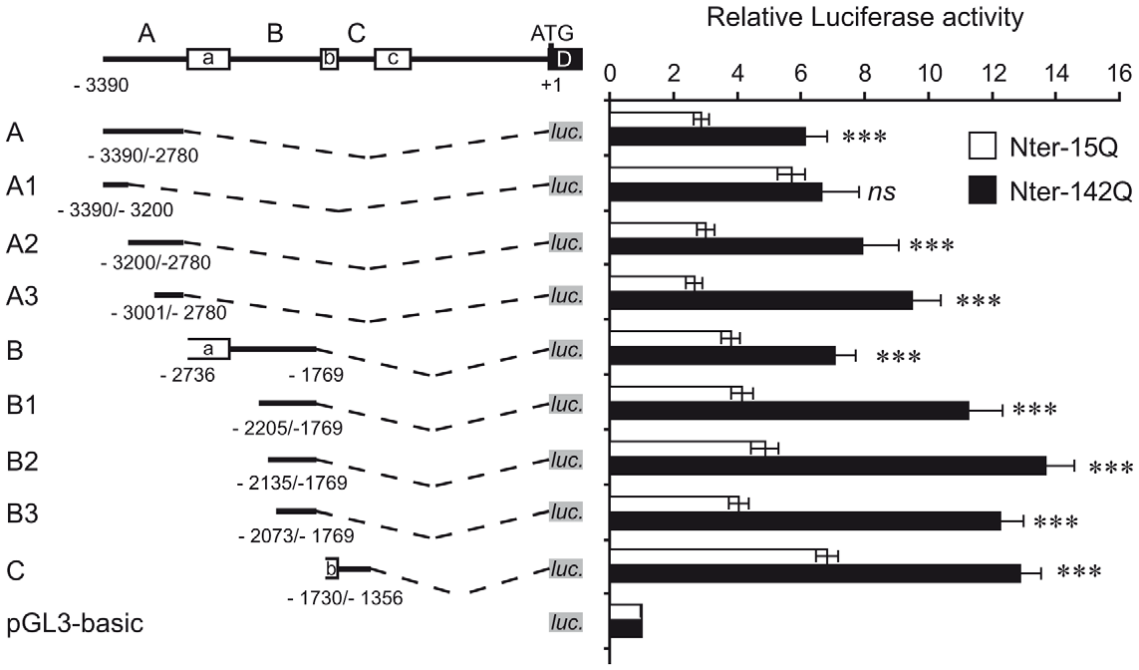
Identification of REST minimal promoter regions regulated by mHtt. On the left, schematic representation of deletion mutants of human REST promoter. Numbers indicate the relative positions with respect to the ATG start codon. The black lines represent the REST promoter regions, the open boxes the three exons (a, b, c) and the dotted lines the deletion between promoter regions and the *luciferase* gene (*luc.*) (grey box). On the right, luciferase activities of deletion mutants. Each deletion construct was cotransfected with Nter-15Q (open bar) or Nter-142Q (solid bar) and beta-galactosidase expression vectors into NG108 cells. Cells were harvested after 6 days of neuronal differentiation to measure luciferase and beta-galactosidase activities. Luciferase activity was normalized to beta-galactosidase activity and expressed as fold increase above the control pGL3-basic. Data are the mean values ± sem of 3 independent experiments performed in triplicate. ***P<0,001; *ns*: non significant, Nter-15Q-expressing cells *vs*. Nter-142Q-expressing cells.

To identify REST promoter regions that are regulated by the Nter of mHtt, we performed sequential promoter deletions and cloned them upstream of the *luciferase* gene into the pGL3-basic vector (Fig. 3). Luciferase vectors were cotransfected with Nter-15Q or Nter-142Q vectors prior to neuronal differentiation of NG108 cells. When compared to Nter-15Q, Nter-142Q led to an increase of luciferase activity for all tested promoter regions, with the exception of region A1. Indeed, we found no significant difference between the luciferase activities driven by the region A1 in Nter-15Q or Nter-142Q neuronal-like cells (Fig. 3). Interestingly, the region A1 contains one putative AP-1 binding site (Supplemental Fig. S1), suggesting that AP-1 transcription factors are not directly involved in REST activation by the Nter of mHtt. Consistently, the promoter regions A2, A3 and C, lacking AP-1 binding sites (Supplemental Fig. S1), were readily activated by Nter-142Q (Fig. 3), likely by the activity of transcriptional factors other than AP-1. Importantly, the results also pointed out three minimal promoter regions, A3, B3 and C, which were sufficient to promote activation by Nter-142Q (Fig. 3).

### Activation of REST by mutant huntingtin does not involve NF-Kappa B

**Figure 4:**
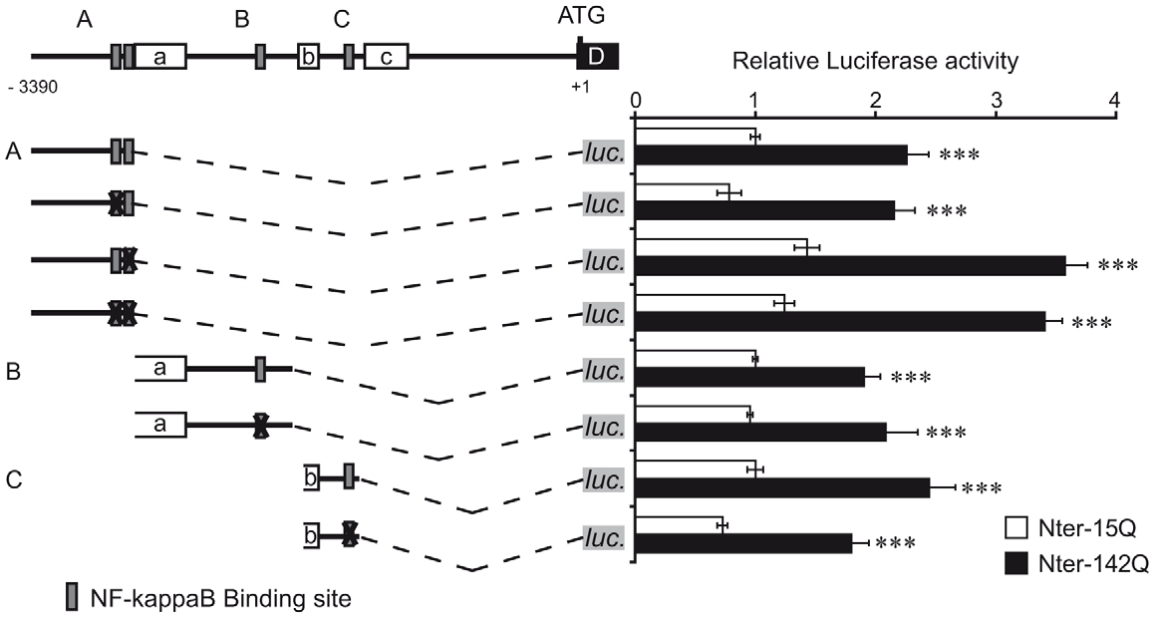
NF-Kappa B is not directly involved in the activation of REST promoter by mHtt. On the left, schematic representations of the three REST promoters A, B and C, with or without mutation(s) in NF-Kappa B binding sites. Numbers indicate the relative positions with respect to the ATG start codon. The black lines represent the REST promoter regions, the open boxes the three exons (a, b, c) and the dotted lines the deletion between promoter regions and the *luciferase* gene (*luc.*) (light grey boxes). Dark grey boxes refer to the location of NF-Kappa B binding sites, while X indicates that these sites were mutated. On the right, luciferase activities of mutated promoters. Each mutated construct was cotransfected with Nter-15Q (open bar) or Nter-142Q (solid bar) and beta-galactosidase expression vectors into NG108 cells. After 6 days of neuronal differentiation, cells were harvested to measure the luciferase and beta-galactosidase activities. Luciferase activity was normalized to beta-galactosidase activity and expressed as fold increase above the activity of construct A, co-expressed with Nter-15Q. Data are the mean values ± sem of 3 independent experiments performed in triplicate. ***P<0,001, Nter-15Q-expressing cells *vs*. Nter-142Q-expressing cells.

Each minimal region, A3, B3 and C, contains putative binding sites for the transcription factor NF-Kappa B (Supplemental Fig. S1). NF-Kappa B binding sites are absent of the promoter region A1, which activity is not increased by Nter-142Q (Supplemental Fig. S1 and Fig. 3). Therefore, NF-Kappa B represented a good candidate to mediate the activation of REST by mHtt. Moreover, several studies have shown that NF-Kappa B activity was impaired in HD. For instance, mouse and cellular models of HD showed an increased nuclear translocation of NF-Kappa B correlating with upregulation of NF-Kappa B target genes [47], [48]. To test this candidate, we generated promoter constructs harbouring mutations within NF-Kappa B binding sites. The core sequence, GGGRNNYYCC, of each NF-Kappa B binding site was replaced by the sequence AAARNNYYTT, which is sufficient to abolish the binding and activity of NF-Kappa B on known NF-Kappa B-regulated promoters [49], [50]. When these constructs were analyzed for their capacity to drive luciferase expression in Nter-15Q or Nter-142Q neuronal-like cells, we found that mutations in NF-Kappa B binding sites did not abolish the activation of REST promoter by Nter-142Q (Fig. 4). The activity of each alternative promoter A, B and C, mutated or not in the NF-Kappa B binding sites, was increased in Nter-142Q compared to Nter-15Q neuronal-like cells. These results indicate that NF-Kappa B was not directly involved in the activation of the REST promoter by the Nter of mHtt in NG108 neuronal-like cells.

### Sp1 and Sp3 regulate the REST promoter in undifferentiated NG108 cells

**Figure 5:**
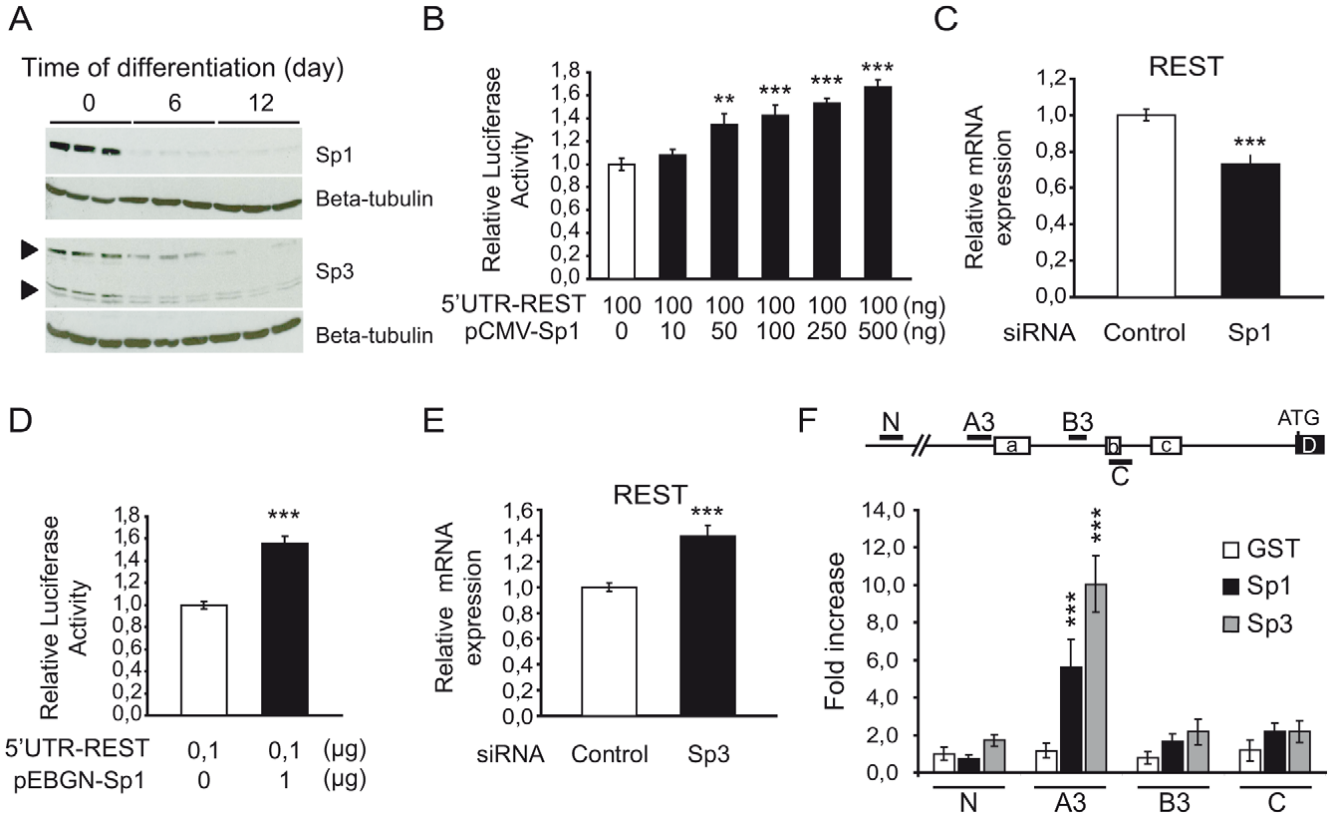
Sp1 and Sp3 regulate REST promoter activity in NG108 cells. (A) Western blot analysis of the level of Sp1 and Sp3 protein in NG108 cells during 12 days of neuronal differentiation. (B) Effect of Sp1 overexpression on REST promoter activity. NG108 cells were cotransfected with 5′UTR-REST vector, increasing amount of Sp1 vector (pCMV-Sp1) and control beta-galactosidase expression vector. Cells were harvested 48 h later to measure luciferase and beta-galactosidase activities. pCMV vector was added to keep the DNA amount constant. Luciferase activity was normalized to beta-galactosidase and expressed as fold increase above the control vector (open bar). Data are the mean values ± sem of 3 independent experiments performed in triplicate. **P<0,01, ***P<0,001, cells transfected with pCMV-Sp1 *vs* cells transfected with empty pCMV vector alone. (C) Effect of Sp1 knockdown on REST mRNA expression. NG108 cells were transfected with specific Sp1 siRNA or control siRNA and analyzed 48 h later for REST mRNA level by quantitative RT-PCR. Each bar represents the mean value ± sem of at least 4 independent experiments performed in triplicate. ***P<0,001, Sp1 siRNA *vs*. control siRNA transfected cells. (D) Effect of a dominant-negative of Sp factors on REST promoter activity. 5′UTR-REST vector was cotransfected with an empty pEBGN vector (open bar) or with dominant-negative Sp1-encoding vector (pEBGN-Sp1) (solid bar) and beta-galactosidase expression vector into NG108 cells. Cells were harvested 48 h later to measure luciferase and beta-galactosidase activities. Luciferase activity was normalized to beta-galactosidase and activity of REST promoter without transfection of pEBGN-Sp1 was set to 1. Data are the mean values ± sem of 3 independent experiments performed in triplicate. ***P<0,001, pEBGN-Sp1 *vs* pEBGN transfected cells. (E) Effect of Sp3 knockdown on REST mRNA expression. NG108 cells were transfected with Sp3 siRNA or control siRNA and analyzed 48 h later for REST mRNA level by quantitative RT-PCR. Each bar represents the mean value ± sem of at least 4 independent experiments performed in triplicate. ***P<0,001, Sp3 siRNA *vs*. control siRNA transfected cells. (F) Sp1 and Sp3 bind REST promoter. ChIP assay was performed to detect Sp1 and Sp3 on REST promoter in NG108 cells using anti-Sp1 and anti-Sp3 antibodies. Sequences covering the regions A, B and C in mouse REST promoter corresponding to the regions A3, B3 and C in human REST promoter were amplified. As a negative control, a sequence 2.0 kb upstream of the region A (N) was amplified. Results are expressed as fold enrichment compared to value obtained after amplification of region N immunoprecipitated with an anti-GST antibody. Each bar represents the mean value ± sem of 6 independent IPs. ***P<0,001.

The three minimal regions, A3, B3 and C, sufficient for REST activation by Nter of mHtt, are GC-rich (70–80%) and contain several putative binding sites for the Sp family of transcription factors. Most of these sites are highly conserved between human, mouse and rat (Supplemental Fig. S1). GC-rich response elements in the REST promoter were previously reported, but their exact roles in REST transcriptional regulation are largely unknown [44], [45], [51]. We performed a series of experiments to determine whether Sp factors could regulate the REST promoter in NG108 cells. We focused on Sp1 and Sp3, whose activities were found to be altered in HD [18], [19], [51]. We found that the levels of Sp1 and Sp3 proteins were high in undifferentiated cells (Fig. 5A and Supplemental Fig. S2). However, Sp1 and Sp3 levels decreased upon neuronal differentiation, correlating with the decreased expression of REST (Fig. 1D). The reduction of Sp1 level was greater than that of Sp3. Sp1 level decreased by more than 90% during neuronal differentiation, while the level of Sp3 protein isoforms were reduced 1,5 to 3 fold (Supplemental Fig. S2). Thus, the amount and ratio of Sp1/ Sp3 proteins declined along neuronal differentiation.

We then explored the role of Sp1 in the regulation of the REST promoter by cotransfecting NG108 cells with the 5′UTR-REST vector and increasing amounts of Sp1 expression vector. As shown in Fig. 5B, overexpression of Sp1 significantly increased REST promoter activity in a dose-dependent manner, suggesting that Sp1 is a transcriptional activator of REST promoter in undifferentiated NG108 cells. To confirm this role, we knocked down the endogenous Sp1 using a specific siRNA. The specificity and efficiency of Sp1 siRNA silencing were confirmed by examining the expression levels of Sp1 and Sp3 by quantitative RT-PCR and western blot (Supplemental Fig. S3). Consistently, Sp1 knockdown significantly reduced the mRNA level of REST in undifferentiated NG108 cells (Fig. 5C). Taken together, these data show that Sp1 positively regulates the transcriptional expression of REST in undifferentiated NG108 cells.

To further study the role of Sp transcription factors in the regulation of REST, we used a more global approach by expressing a dominant-negative of Sp factors (Sp-DN). Sp-DN is the DNA binding domain of Sp1, which binds to GC-rich response elements, and can thus compete with endogenous Sp factors on promoters [37]. Expression and interfering activity of Sp-DN in NG108 cells were verified by using the MLN64 promoter, known to be positively regulated by Sp1 [52] (Supplemental Fig. S4). Surprisingly, when we cotransfected NG108 cells with 5′UTR-REST and Sp-DN vectors, the overexpression of Sp-DN led to an increased activity of REST promoter in undifferentiated NG108 cells (Fig. 5D). This result suggests that Sp-DN prevents the binding of Sp factor having a repressive effect on REST promoter. Therefore, we hypothesized that the expression level of REST in undifferentiated NG108 is controlled by the competition between the activating activity of Sp1 and the repressive activity of another Sp factor.

Sp3 has a high homology to Sp1, reaching more than 90% in the DNA-binding domains. Sp3 acts both as activator and repressor of transcription. Interestingly, a number of studies showed that Sp3 can repress Sp1-mediated transactivation of promoters [53], [54]. To determine if Sp3 repressed the transcriptional activity of the REST promoter, we knocked down the endogenous Sp3 using siRNA and analyzed the level of REST mRNA. The specificity and efficiency of Sp3 siRNA knockdown were confirmed by examining the expression levels of Sp3 and Sp1 by quantitative RT-PCR and western blot (Supplemental Fig. S3). Consistent with our hypothesis, Sp3 knockdown led to an increased level of REST mRNA (Fig. 5E). Taken together, our data show that both Sp1 and Sp3 regulate REST promoter in undifferentiated NG108 cells and that Sp1 acts as an activator and Sp3 as a repressor.

### Sp1 and Sp3 directly bind to the REST promoter in NG108 cells

To assess whether the regulation of REST by Sp1 and Sp3 is direct or indirect, we performed a ChIP experiment using Sp1 or Sp3 specific antibodies. Both Sp1 and Sp3 were specifically bound to the A3 region of the mouse REST promoter in undifferentiated NG108 cells, but were absent from B3 and C regions, and from a control genomic DNA fragment located 2,0 kb upstream of the REST A3 promoter region (Fig. 5F). These results indicate that Sp1 and Sp3 regulate transcription of *Rest* gene by acting directly on its promoter.

### REST activation by mutant huntingtin is mediated by Sp1 in NG108 neuronal-like cells

**Figure 6:**
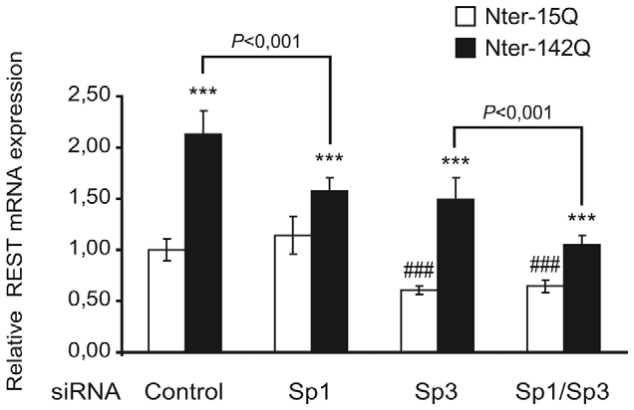
Knockdown of endogenous Sp1 decreases mHtt-mediated REST activation. NG108 cells were cotransfected with Nter-15Q or Nter-142Q vectors and Sp1 or/and Sp3 siRNA. After 6 days of neuronal differentiation, REST mRNA level was analyzed by quantitative RT-PCR. Each bar represents the mean values ± sd of at least 2 independent experiments performed in triplicate. ***P<0,001, Nter-15Q neuronal-like cells *vs*. Nter-142Q neuronal-like cells; ###*P*<0,001, Nter-15Q neuronal-like cells transfected with control siRNA *vs* Nter-15Q neuronal-like cells transfected with Sp3 siRNA or Sp1/Sp3 siRNA.

To clarify the role of Sp1 and Sp3 in the transcriptional activation of REST by Nter of mHtt, we knocked down Sp1 and/or Sp3 in Nter-15Q and Nter-142Q neuronal-like cells. The specificity and efficiency of Sp1 siRNA, Sp3 siRNA and Sp1/Sp3 siRNAs silencing in Nter-15Q and Nter-142Q neuronal-like cells were assessed by western blot and quantitative RT-PCR (Supplemental Fig. S5). The knockdown of Sp1 in Nter-15Q neuronal-like cells did not modify the mRNA level of REST (Fig. 6), suggesting that Sp1 does not regulate the basal activity of the REST promoter in NG108 neuronal-like cells. However, in Nter-142Q neuronal-like cells, the inhibition of Sp1 decreased the Nter-142Q-mediated activation of REST by about 60% (Fig. 6). This result indicates that REST activation by Nter-142Q in NG108 neuronal-like cells is mediated by Sp1. The inhibition of Sp3 significantly decreased REST expression similarly in Nter-15Q and Nter-142Q neuronal-like cells (Fig. 6), suggesting that Sp3 is switched from a repressor in undifferentiated NG108 cells to an activator of REST transcription in NG108 neuronal-like cells. Moreover, the result indicates that the activating activity of Sp3 is required for the basal REST expression in neuronal-like cells.

Consistent with the above results, the double knockdown of Sp1 and Sp3 in neuronal-like cells corroborated their role in REST expression (Fig. 6). In Nter-15Q neuronal-like cells, Sp1/Sp3 double knockdown reduced REST expression at a level comparable to that of Sp3 knockdown alone (Fig. 6), thereby confirming that Sp3 is majorly involved in the basal REST expression in these cells. In Nter-142Q neuronal-like cells, the level of REST expression was much more reduced by Sp1/Sp3 double knockdown than by Sp3 or Sp1 knockdown alone (Fig. 6). This confirms that REST upregulation by Nter-142Q in NG108 neuronal-like cells is predominantly mediated by Sp1, while a contribution of Sp3 cannot be excluded.

To further investigate the role of Sp1 and Sp3 in mHtt-mediated upregulation of REST, we performed ChIP experiments from whole brains of 12 week-old R6/2 mice. As in the striatum, REST expression was increased in the whole brain of R6/2 mice, when compared to control mice (Supplemental figure S6). Although Sp1 and Sp3 signals were not significantly different between R6/2 and control mice, our data showed that Sp1 and Sp3 were significantly enriched at the A3 region of REST promoter in R6/2 brains, but not in control brains (Supplemental figure S6), indicating that Sp factors might contribute to REST RNA upregulation in R6/2 mice.

Therefore, our results from NG108 neuronal-like cells indicate that mHtt-mediated upregulation of REST predominantly involves Sp1 activity, and possibly Sp3. Our data suggest that this mechanism also occurs in R6/2 mice.

## Discussion

Using cellular and mouse models of HD, we show that REST mRNA is significantly increased in neurons expressing mHtt fragments. Our data provide evidence that Sp1 and Sp3 transcription factors regulate REST expression in undifferentiated NG108 cells and in NG108 cells differentiated into neuronal-like cells, and that Sp factors transcriptional roles change during the switch from proliferation to neuronal differentiation. Our study supports a role for Sp1, and likely Sp3, in the mHtt-mediated upregulation of REST in NG108 neuronal-like cells and in R6/2 mice, thus suggesting that the effect of mHtt on REST function is in part indirect.

The mechanism underlying HD pathogenesis is not yet fully understood. However, numerous studies provide evidence that part is related to transcriptional dysregulations, which progressively worsen during the course of the disease [4], [55], [56]. The activation of REST, a master regulator of neuronal genes [27], [28], has been implicated in this process. Zuccato *et al.* [30], [31] studied the mechanism of REST activation at the RNA and protein levels. While no significant increase of REST RNA levels was found in HD cellular and mouse models, they demonstrated that loss of Htt function leads to nuclear translocation of REST in various HD cellular models, thereby causing a REST-mediated repression of several neuronal genes and contributing to neuronal dysfunction. Here, we show that the level of REST mRNA is significantly increased in our HD neuronal-like model as well as in HD R6/2 mice. Our results are consistent with the study of Smith *et al.* [33] showing an increased level of REST protein in HD R6/1 mice. The discrepancy might be due to the use of different methodologies: Zuccato and collaborators performed a semi-quantitative method to measure REST RNA level, while we used the accurate quantitative RT-PCR. Our data are supported by the fact that Nter-142Q activates the REST promoter in the luciferase gene reporter assay performed in NG108 neuronal-like cells. Another explanation for the discrepancy may lie in the HD models *per se*. Zuccato *et al.* [31] mainly studied HD models expressing full length mHtt, while the HD models analyzed by our group express Nter fragments of mHtt, suggesting that the full length form and fragments of mHtt activate REST by different mechanisms. Packer *et al* [34] recently showed that MiR-9, a microRNA that regulates REST expression level, is downregulated in HD and may account for the observed increase of REST expression. Thus, mHtt and its Nter fragments could trigger the activation of REST through 3 different mechanisms: by increasing REST transcription, decreasing microRNA regulation and increasing REST nuclear translocation.

Earlier studies have shown that the REST gene contains 3 alternative promoters, characterized by different levels of activity [43], [44], [45]. It was shown that the activities of promoters A and B were predominant, promoter A being the most active, while the activity of promoter C was negligible in both proliferating neural and non-neural cells [44], [45]. Our experiments based on the use of luciferase constructs containing each of the 3 REST promoters show that the activities of the 3 alternative promoters are increased by Nter-mHtt in NG108 differentiated into neuronal-like cells (Fig. 3). The significant activity of promoter C in our experimental system compared to previous studies might be due to differences in the reporter constructs. In our study, promoter C (construct C in Fig. 3) was placed immediately upstream of the *luciferase* reporter gene, while constructs used in previous studies had exon C between the promoter and reporter gene, which is proposed to contain a repressor region [44].

Bioinformatics analysis revealed that the REST promoters contained several putative binding sites for AP-1, NF-Kappa B and Sp factors. Using deletion constructs, we delineated the Nter-mHtt-responsive minimal regions within each promoter and excluded a direct implication of the AP-1 element located in promoter A (Fig. 3). The NF-Kappa B binding elements were exclusively present within the minimal promoter regions. However, mutations of these sites showed that none of the NF-Kappa B elements were involved in the Nter-mHtt-mediated upregulation of REST in NG108 neuronal-like cells (Fig. 4).

The Nter-mHtt-responsive minimal regions also contained several GC-boxes, which are known to be potential Sp binding elements. Using luciferase reporter and gel-mobility shift assays, Kojima *et al.* [45] showed that GC-boxes within promoter A were functional in the NIH3T3 fibroblasts and the Neuro2a neuroblastoma cell line. Since no difference was found between neuronal and non-neuronal cell types in proliferating conditions, they suggested that the level of REST gene expression is not determined by transcription per se in a cell-specific manner. The authors identified neither the factors that bind these GC-boxes, nor the mechanism of REST regulation. Our study extends Kojima's results by providing several lines of evidence for a specific role of Sp factors in the regulation of REST in neuronal and non-neuronal cells. First, using RNAi-based and overexpression experiments, we demonstrate that Sp factors, Sp1 and Sp3, regulate REST promoter activity in NG108 undifferentiated and differentiated into neuronal-like cells (Fig. 5 and 6). Second, our ChIP data support that promoter A is the main target of Sp factors, as enrichment of Sp1 and Sp3 is significant at promoter A in NG108 cells and in R6/2 brains (Fig. 5F and S6). Third, taking advantage of our cellular system where the activity of REST promoter was properly compared in a single cell type in undifferentiated/proliferating *versus* differentiated/non-proliferating conditions, we specified the role of Sp1 and Sp3 in the downregulation of REST expression during neuronal differentiation. Our results provide evidence that Sp1 and Sp3 exert an antagonistic effect on REST regulation in undifferentiated NG108 cells, Sp1 being an activator and Sp3 an inhibitor (Fig. 5). The resulting global effect of Sp1 and Sp3 activities ensures high REST expression in undifferentiated NG108 cells. Moreover, we show that upon neuronal differentiation, the amount and ratio of Sp1 and Sp3 decrease (as discussed below) and correlate with a reduction of the basal activity of the REST promoter.

The complex interplay of Sp1 and Sp3 in REST regulation can be rationally interpreted in the light of earlier studies. Sp3 is known to be a versatile transcription factor [53], [54], which either suppresses promoter activation by Sp1 or, at the opposite, cooperates with Sp1 on promoters. The outcome appears to depend on the cellular and gene promoter context. Our data suggest that Sp3 behaves as an activator and a repressor on the REST promoter in NG108 cells, depending of the differentiation state. The RNAi experiments indeed show that Sp3 behaves as an activator of REST in neuronal-like cells expressing the Nter of wildtype Htt (Fig. 6). It is believed that the relative ratios between Sp factors, post-translational modifications or specific interactions with other transcription factors modulate the transcriptional competence of Sp factors at a given promoter in a cell- or signal-specific manner [53], [54]. Our western-blotting and RNAi experiments show that Sp1 protein was strongly decreased during neuronal differentiation. Sp3 proteins were also reduced during neuronal differentiation, but less than Sp1 proteins (Fig. 5A). This indicates that both the relative ratio and global amounts of Sp1 and Sp3 isoforms change upon neuronal differentiation, which likely contribute to modify the transcriptional role of Sp3. In contrast to Sp1, Sp3 lacks the ability to transactivate synergistically *via* two or more Sp sites. In consequence, it has been proposed that if Sp3 replaces Sp1 on promoters containing two of more Sp sites, the transactivation effect of Sp3 would be weaker than that of Sp1, resulting in a net repression of the initial Sp1-mediated transactivation [53]. We suggest that this situation might occur at the REST promoter upon neuronal differentiation: decrease of Sp1/Sp3 ratio would contribute to down regulation of REST during neuronal differentiation by reducing the basal activity of the promoter. This mechanism provides for the first time a molecular clue to the transcriptional repression of REST upon neuronal differentiation.

Our ChIP and RNAi-based results support a role for Sp1, and probably Sp3, in the mHtt-mediated upregulation of REST in NG108 neuronal-like cells (Fig. 6), suggesting that mHtt increases Sp1 and Sp3 activity at the REST promoter in neurons. Several studies reported that Sp1 proteins are moderately but significantly increased (by about 2-fold) in the brain of HD mice and in striatal or neuronal cells expressing mHtt [20], [22], [51]. Accordingly, overall DNA binding by Sp1 was increased in R6/2 mouse brain nuclear extracts [20]. We asked whether Sp1 or Sp3 increased activity in NG108 neuronal-like cells expressing Nter-mHtt could be explained by an increase in protein level, but we could not detect a significant change of Sp1 or Sp3 level in these cells (data not shown). This is likely due to the transfection efficiency, which did not allow expression of the mHtt fragment in all cells. In these conditions, a moderate (2-fold) increase of Sp1 or Sp3 proteins would be very difficult to quantify. A growing number of studies have shown that the activities of Sp factors are regulated in signal- and tissue-specific manners and play a critical role in the regulation of inducible gene expression, thus controlling cell homeostasis as well as major physiological processes, including growth control, differentiation or survival. For instance, it has been shown that oxidative stress induces Sp1 and Sp3 transcriptional activities in cortical neurons [51]. Similarly, treatment of neurons with excitotoxic drugs leading to oxidative stress, including the mitochondrial toxin 3-NP or the glutamate receptor agonist kainic acid, results in induction of Sp1/Sp3 activities [51], [57]. In HD mice and patients, oxidative stress, which is in part mediated by excitotoxic mechanisms, has been shown to be increased [58], suggesting that Sp1/Sp3 activation might be driven by oxidative stress in HD. Interestingly, treatments inducing oxidative stress, including kainic acid and ischemia, derepress REST expression in various types of neurons [15], [59], [60]. It is therefore tempting to speculate that the detrimental upregulation of REST in HD neurons, which is expected to impair the identity of neurons, involves Sp1 and Sp3 functions and may be oxidative stress-mediated. This situation might be reminiscent to what we observed previously in the context of a SCA7 retinal mouse model, where alteration of the neuronal differentiation program by mutant ataxin-7 appeared to be stress-induced [15], [46], [61].

In conclusion, our study together with earlier reports suggest that mHtt fragments trigger a pathogenic cascade involving activation of Sp1 and Sp3, which in turn upregulates REST that represses neuronal genes in HD. Supporting this view, the reduction of Sp1 function in HD mice and the reduction of REST in HD cells have both proven to be beneficial [21], [22], [29].

## Supporting Information

Figure S1 Alignment of the human, mouse and rat REST gene promoters. The nucleotide sequences of the three exons a, b and c and their respective upstream promoters are shown. Nucleotide numbers on the right of each lane correspond to the human promoter with respect to the ATG start codon. Conserved nucleotides are marked with asterisk. Sequences in bold correspond to three exons. The putative binding sites for AP-1 (underlined), NF-Kappa B (grey boxes) and Sp factors (GC-box in black) are indicated.
(17.10 MB TIF)

Figure S2 Relative Sp1 and Sp3 protein levels in NG108 cells during 12 days of neuronal differentiation. Sp1 and the long and two short isoforms of Sp3 (L-Sp3 and S-Sp3, respectively) protein levels were quantified on western blots shown in figure 6A and normalized using beta-Tubulin as loading control. *P<0,05; **P<0,01 and ***P<0,001.
(0.14 MB TIF)

Figure S3 Effect of Sp1 and Sp3 knockdown on Sp1 and Sp3 expression levels in undifferentiated NG108 cells. NG108 cells were transfected with specific Sp1 siRNA, Sp3 siRNA or control siRNA and analyzed 48 h later for Sp1 and Sp3 mRNA levels by quantitative RT-PCR (A) and protein levels by western blot (B). Each bar represents the mean value ± sem of at least 4 independent experiments performed in triplicate. *P<0,05, Sp1 siRNA or Sp3 siRNA transfected cells vs. control siRNA transfected cells.
(0.56 MB TIF)

Figure S4 A dominant-negative of Sp factors downregulated the promoter of MLN64 gene. The pGL3-0.2-MLN64 vector, which expresses the luciferase gene driven by the MLN64 promoter [51] was cotransfected with an empty pEBGN vector (open bar) or with dominant-negative Sp1-encoding vector (pEBGN-Sp1) (solid bar) and beta-galactosidase expression vector into NG108 cells. Cells were harvested 48 h later to measure luciferase and beta-galactosidase activities. Luciferase activity was normalized to beta-galactosidase and activity of MLN64 promoter without transfection of pEBGN-Sp1 was set as 1. Data are the mean values ± sem of 3 independent experiments performed in triplicate. ***P<0,001, pEBGN-Sp1 vs pEBGN transfected cells.
(0.14 MB TIF)

Figure S5 Effect of Sp1 and Sp3 knockdown on Sp1 and Sp3 expression levels in NG108 cells differentiated into neurons. NG108 cells were cotransfected with Nter-15Q or Nter-142Q vectors and with Sp1 siRNA, Sp3 siRNA, Sp1/sp3 siRNAs or control siRNA. Cells were differentiated into neuronal-like cells for 6 days prior to analysis. (A) Sp1 mRNA level (left panel) and Sp3 mRNA level (right panel) were analyzed by quantitative RT-PCR. Each bar represents the mean values ± sd of a single representative experiment performed in triplicate. ***P<0,001, neuronal cells transfected with control siRNA vs neuronal cells transfected with Sp1 or Sp3 siRNAs. (B) Sp1 protein level were analyzed by western blot using beta-Tubulin as loading control. (C) Sp3 protein level was analyzed by western blot using beta-Tubulin as loading control.
(3.37 MB TIF)

Figure S6 Sp1 and Sp3 bind REST promoter in the brain of 12 week-old R6/2 mice. (A) mRNA level of REST in the whole brain of 12-week-old wild-type (WT) (open bar) and R6/2 mice (solid bar). mRNA level was determined by quantitative RT-PCR. Each bar represents the mean value ± sem, *P<0,05 (WT: n = 4; R6/2: n = 4). (B) ChIP assay was performed to detect Sp1 and Sp3 on REST promoter in 12 week-old R6/2 and wild-type (WT) brains using anti-Sp1 and anti-Sp3 antibodies. Sequences covering the regions A in mouse REST promoter corresponding to the region A3 in human REST promoter was amplified. As a negative control, a sequence 2.0 kb upstream of the region A (N) was amplified. Results are expressed as fold enrichment compared to value obtained after amplification of the regions treated with no antibody. Each immunoprecipitation was performed in triplicate, corresponding to 3 different mice. The bars represent the mean value obtained from triplicate +/− sem; * P<0,05.
(0.16 MB TIF)

Table S1 Oligonucleotide primers used for DNA amplification of human NRSF promoter regions.
(0.05 MB DOCX)

Table S2 Oligonucleotide primers used for direct mutagenesis of the putative NF-KappaB binding sites of human NRSF promoter regions named A, B and C.
(0.05 MB DOCX)

Table S3 Oligonucleotide primers used for gene expression analysis by quantitative real-time reverse transcription-PCR.
(0.04 MB DOCX)

Table S4 Oligonucleotide primers used for chromatin immunoprecipitation analysis of mouse NRSF promoter by quantitative real-time reverse transcription-PCR.
(0.05 MB DOCX)
