# Supplementary figures and images for: Transcriptional Activation of REST by Sp1 in Huntington's Disease Models
Source: PLoS One. 2010 Dec 14;5(12):e14311. doi: 10.1371/journal.pone.0014311 (PMC3001865; doi:10.1371/journal.pone.0014311)

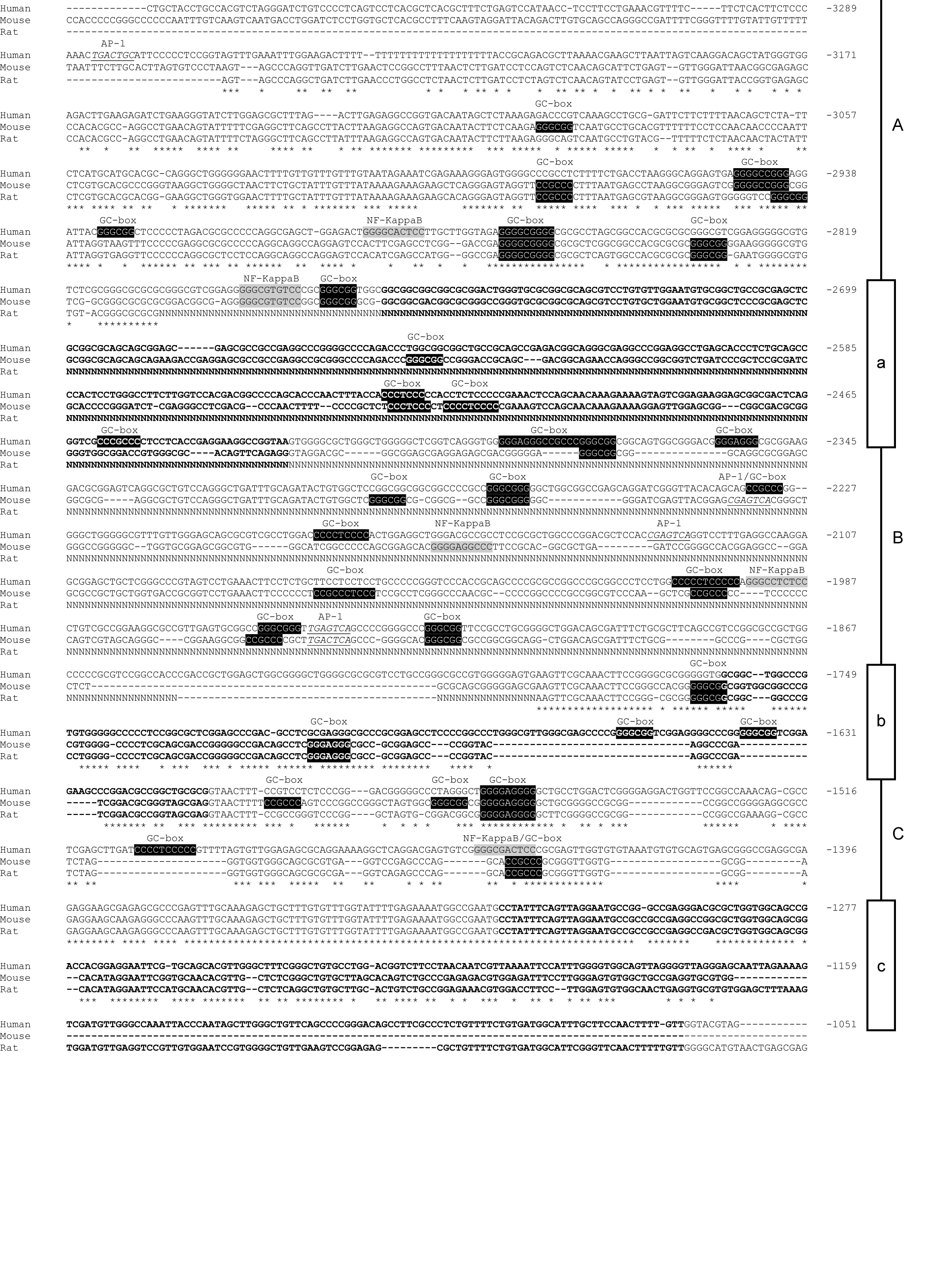

Supplement: Figure S1 — Alignment of the human, mouse and rat REST gene promoters. The nucleotide sequences of the three exons a, b and c and their respective upstream promoters are shown. Nucleotide numbers on the right of each lane correspond to the human promoter with respect to the ATG start codon. Conserved nucleotides are marked with asterisk. Sequences in bold correspond to three exons. The putative binding sites for AP-1 (underlined), NF-Kappa B (grey boxes) and Sp factors (GC-box in black) are indicated. (17.10 MB TIF) [file pone.0014311.s001.tif]

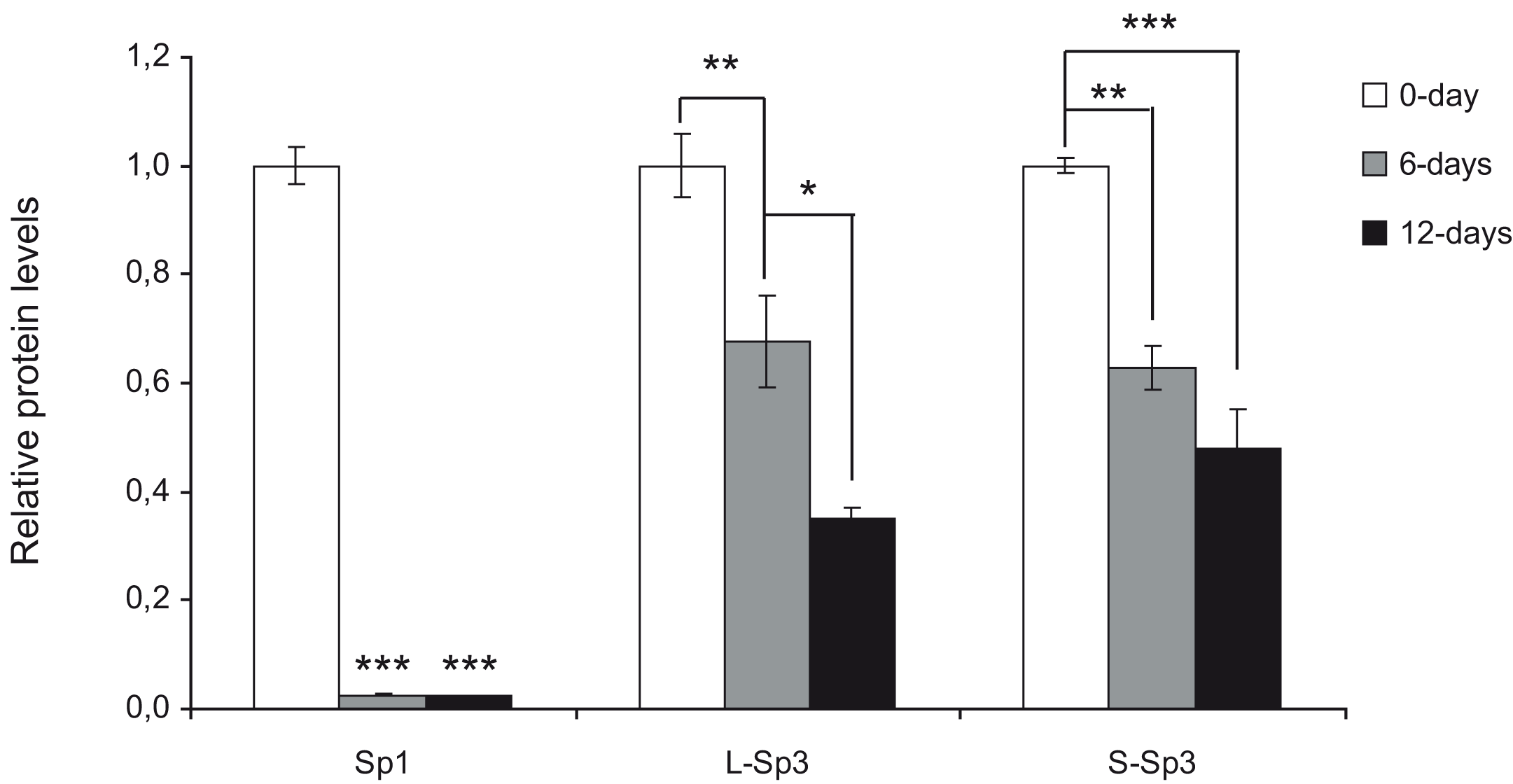

Supplement: Figure S2 — Relative Sp1 and Sp3 protein levels in NG108 cells during 12 days of neuronal differentiation. Sp1 and the long and two short isoforms of Sp3 (L-Sp3 and S-Sp3, respectively) protein levels were quantified on western blots shown in figure 6A and normalized using beta-Tubulin as loading control. *P<0,05; **P<0,01 and ***P<0,001. (0.14 MB TIF) [file pone.0014311.s002.tif]

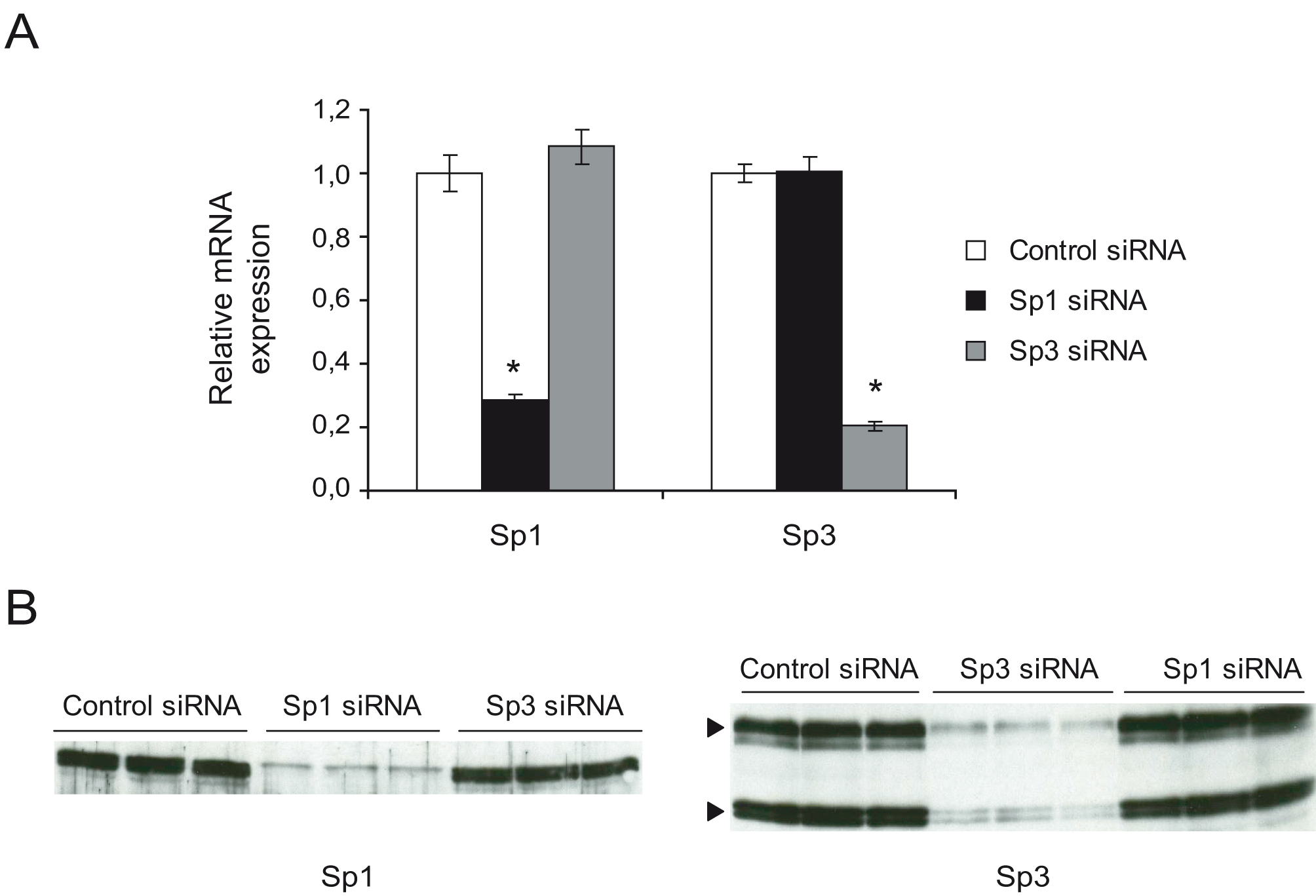

Supplement: Figure S3 — Effect of Sp1 and Sp3 knockdown on Sp1 and Sp3 expression levels in undifferentiated NG108 cells. NG108 cells were transfected with specific Sp1 siRNA, Sp3 siRNA or control siRNA and analyzed 48 h later for Sp1 and Sp3 mRNA levels by quantitative RT-PCR (A) and protein levels by western blot (B). Each bar represents the mean value ± sem of at least 4 independent experiments performed in triplicate. *P<0,05, Sp1 siRNA or Sp3 siRNA transfected cells vs. control siRNA transfected cells. (0.56 MB TIF) [file pone.0014311.s003.tif]

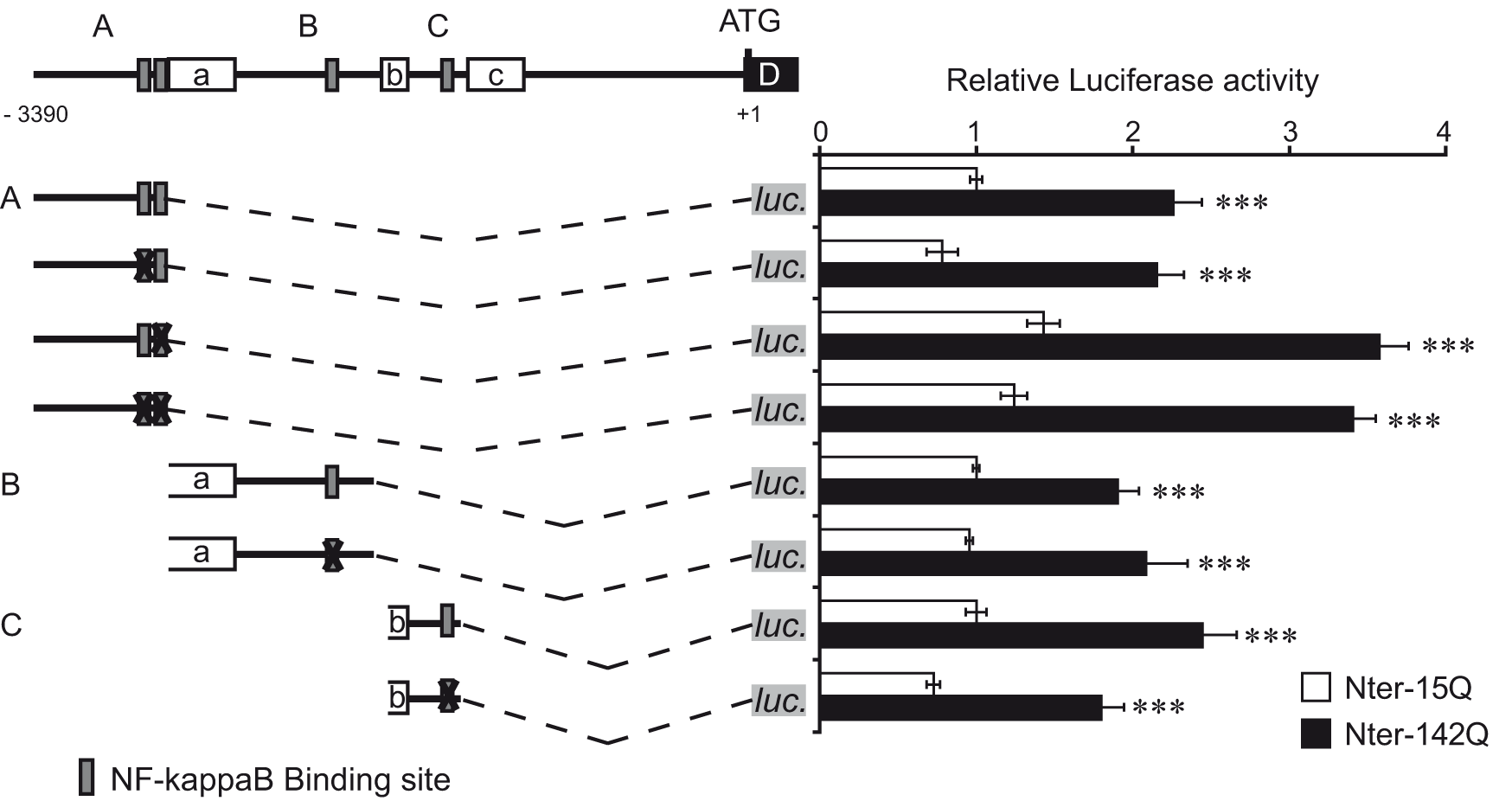

Supplement: Figure S4 — A dominant-negative of Sp factors downregulated the promoter of MLN64 gene. The pGL3-0.2-MLN64 vector, which expresses the luciferase gene driven by the MLN64 promoter [51] was cotransfected with an empty pEBGN vector (open bar) or with dominant-negative Sp1-encoding vector (pEBGN-Sp1) (solid bar) and beta-galactosidase expression vector into NG108 cells. Cells were harvested 48 h later to measure luciferase and beta-galactosidase activities. Luciferase activity was normalized to beta-galactosidase and activity of MLN64 promoter without transfection of pEBGN-Sp1 was set as 1. Data are the mean values ± sem of 3 independent experiments performed in triplicate. ***P<0,001, pEBGN-Sp1 vs pEBGN transfected cells. (0.14 MB TIF) [file pone.0014311.s004.tif]

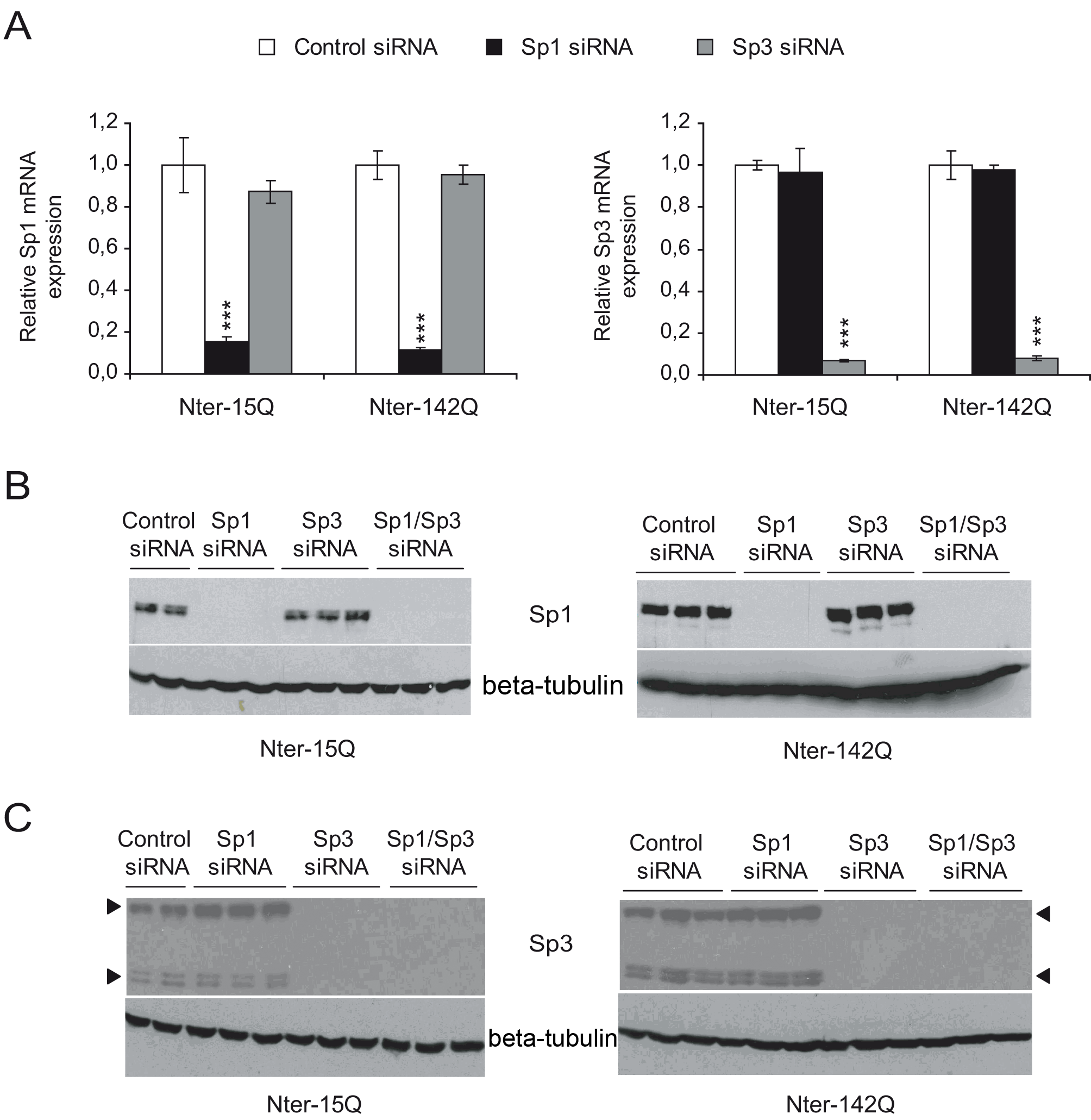

Supplement: Figure S5 — Effect of Sp1 and Sp3 knockdown on Sp1 and Sp3 expression levels in NG108 cells differentiated into neurons. NG108 cells were cotransfected with Nter-15Q or Nter-142Q vectors and with Sp1 siRNA, Sp3 siRNA, Sp1/sp3 siRNAs or control siRNA. Cells were differentiated into neuronal-like cells for 6 days prior to analysis. (A) Sp1 mRNA level (left panel) and Sp3 mRNA level (right panel) were analyzed by quantitative RT-PCR. Each bar represents the mean values ± sd of a single representative experiment performed in triplicate. ***P<0,001, neuronal cells transfected with control siRNA vs neuronal cells transfected with Sp1 or Sp3 siRNAs. (B) Sp1 protein level were analyzed by western blot using beta-Tubulin as loading control. (C) Sp3 protein level was analyzed by western blot using beta-Tubulin as loading control. (3.37 MB TIF) [file pone.0014311.s005.tif]

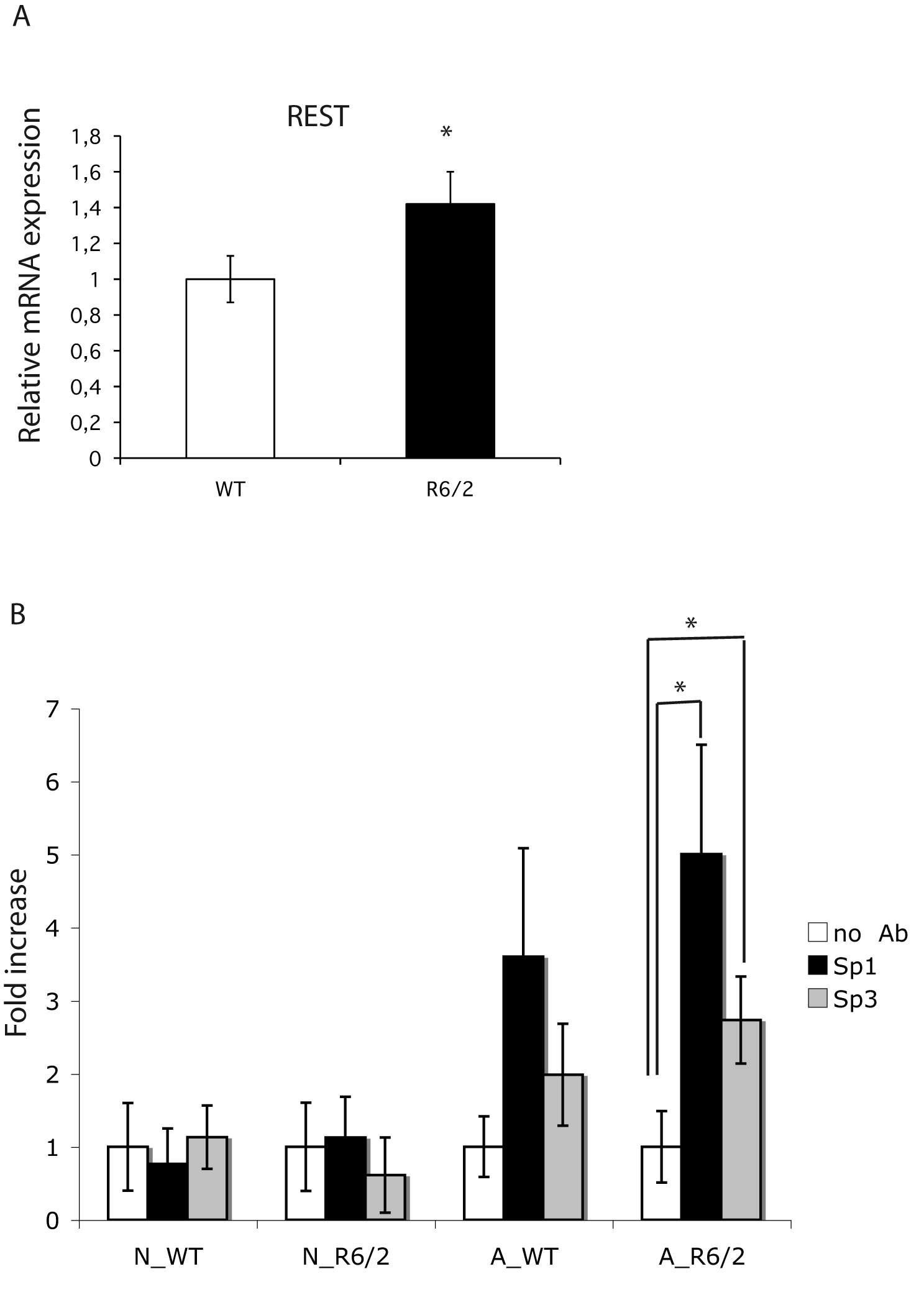

Supplement: Figure S6 — Sp1 and Sp3 bind REST promoter in the brain of 12 week-old R6/2 mice. (A) mRNA level of REST in the whole brain of 12-week-old wild-type (WT) (open bar) and R6/2 mice (solid bar). mRNA level was determined by quantitative RT-PCR. Each bar represents the mean value ± sem, *P<0,05 (WT: n = 4; R6/2: n = 4). (B) ChIP assay was performed to detect Sp1 and Sp3 on REST promoter in 12 week-old R6/2 and wild-type (WT) brains using anti-Sp1 and anti-Sp3 antibodies. Sequences covering the regions A in mouse REST promoter corresponding to the region A3 in human REST promoter was amplified. As a negative control, a sequence 2.0 kb upstream of the region A (N) was amplified. Results are expressed as fold enrichment compared to value obtained after amplification of the regions treated with no antibody. Each immunoprecipitation was performed in triplicate, corresponding to 3 different mice. The bars represent the mean value obtained from triplicate +/− sem; * P<0,05. (0.16 MB TIF) [file pone.0014311.s006.tif]
