# Supplementary material for: Transcriptional Activation of REST by Sp1 in Huntington's Disease Models
Source: PLoS One. 2010 Dec 14;5(12):e14311. doi: 10.1371/journal.pone.0014311 (PMC3001865; doi:10.1371/journal.pone.0014311)
Supplement: Table S1 — Oligonucleotide primers used for DNA amplification of human NRSF promoter regions. (0.05 MB DOCX) [file pone.0014311.s007.docx]

| **Construct name** | **Forward primers** | **Reserve primers** |
| --- | --- | --- |
| 5'UTR-NRSF | 5'-ctgctacctgccacgtct-3' | 5’-gtggccataactgtattctga-3’ |
| A | 5'-ctgctacctgccacgtct-3' | 5'-gggacacgccccctccgacg-3' |
| A3 | 5'-tcgagaaagggagtggggcc-3' | 5'-gggacacgccccctccgacg-3' |
| B | 5'-agcgtcctgtgttggaatgt-3' | 5'-cgccccggaagtttgcga-3' |
| B1 | 5'-cagcgcgtcgcctggac-3' | 5'-cgccccggaagtttgcga-3' |
| B2 | 5'-accgagtcaggtcctttgagg-3' | 5'-cgccccggaagtttgcga-3' |
| B3 | 5'-ctctgcttcctcctcctgcc-3' | 5'-cgccccggaagtttgcga-3' |
| B4 | 5'-gccgttgagtgcggccg-3 | 5'-cgccccggaagtttgcga-3' |
| C | 5'-gctcggagcccgacgcct-3' | 5'-aaagcagctctttgcaaactc-3' |
