# Supplementary material for: Transcriptional Activation of REST by Sp1 in Huntington's Disease Models
Source: PLoS One. 2010 Dec 14;5(12):e14311. doi: 10.1371/journal.pone.0014311 (PMC3001865; doi:10.1371/journal.pone.0014311)
Supplement: Table S2 — Oligonucleotide primers used for direct mutagenesis of the putative NF-KappaB binding sites of human NRSF promoter regions named A, B and C. (0.05 MB DOCX) [file pone.0014311.s008.docx]

| **Construct name** | **Forward primers** | **Reverse primers** |
| --- | --- | --- |
| **A (first site)** | 5'-aggcgagctggagact**aaa**gcact**tt**ttgcttggtagaggggc-3' | 5'-gcccctctaccaagcaa**aa**agtgc**ttt**agtctccagctcgcct-3' |
| **A (second site)** | 5'-gcgcgggcgtcggagg**aaa**cgtgt**tt**cctcgagatctgcg-3' | 5'-cgcagatctcgagg**aa**acacg**ttt**cctccgacgcccgcgc-3' |
| **B** | 5'-tggccccctccccca**aaa**cctct**tt**ctgtcgccggaaggc-3' | 5'-gccttccggcgacag**aa**agagg**ttt**tgggggagggggcca-3' |
| **C** | 5'-ggctcaggacgagtgtcg**aaa**cgact**tt**cgcgagttggtgtg-3' | 5'-cacaccaactcgcg**aa**agtcg**ttt**cgacactcgtcctgagcc-3' |

The putative NF-KappaB binding sites are underlined and introduced mutations are indicated in bold.
