# Supplementary material for: Transcriptional Activation of REST by Sp1 in Huntington's Disease Models
Source: PLoS One. 2010 Dec 14;5(12):e14311. doi: 10.1371/journal.pone.0014311 (PMC3001865; doi:10.1371/journal.pone.0014311)
Supplement: Table S3 — Oligonucleotide primers used for gene expression analysis by quantitative real-time reverse transcription-PCR. (0.04 MB DOCX) [file pone.0014311.s009.docx]

|  | **Foward primers** | **Reserve primers** |
| --- | --- | --- |
| **NRSF** | 5'-ttccccaggaaagtctacacc-3' | 5'-catgctgattagaggccacat-3' |
| **Synaptophysine** | 5'-tgacttcaggactcaacacctc-3' | 5'-caggagctggttgcttttct-3' |
| **Sp1** | 5'-tgggtacttcagggatccag-3' | 5'-tccttctccacctgctgtct-3' |
| **Sp3** | 5'-tgctgacagtcctgcagata-3' | 5'-ggtccccttcttcatctacc-3' |
