# Supplementary material for: Transcriptional Activation of REST by Sp1 in Huntington's Disease Models
Source: PLoS One. 2010 Dec 14;5(12):e14311. doi: 10.1371/journal.pone.0014311 (PMC3001865; doi:10.1371/journal.pone.0014311)
Supplement: Table S4 — Oligonucleotide primers used for chromatin immunoprecipitation analysis of mouse NRSF promoter by quantitative real-time reverse transcription-PCR. (0.05 MB DOCX) [file pone.0014311.s010.docx]

|  | **Foward primers** | **Reserve primers** |
| --- | --- | --- |
| **A3** | 5'-GAAGCTCAGGGAGTAGGTTCCG-3' | 5'-GGACACGCCCCCTCGCCGTC-3' |
| **B3** | 5'-ACCGCGGTCCTGAAACTT-3' | 5'-GTGCCCCGGGCTGAGTCAAG-3' |
| **C** | 5'-TCGCAGCGACCGGGGGCC-3' | 5'-GCTGCCCACCACCCTAGA-3' |
| **N** | 5'-ACTGGAGAGACAGCTCAGTGG-3' | 5'-TTTCTTTCCTGTGTTGTTTGTG-3' |

The region, named N, localized 2000 bp upstream the region A3 was probed as a negative control.
